# Persistence of senescent prostate cancer cells following prolonged neoadjuvant androgen deprivation therapy

**DOI:** 10.1371/journal.pone.0172048

**Published:** 2017-02-24

**Authors:** Michael L. Blute, Nathan Damaschke, Jennifer Wagner, Bing Yang, Martin Gleave, Ladan Fazli, Fangfang Shi, E. Jason Abel, Tracy M. Downs, Wei Huang, David F. Jarrard

**Affiliations:** 1 Department of Urology University of Wisconsin School of Medicine and Public Health, Highland Ave, Madison, Wisconsin, United States of America; 2 Vancouver Prostate Centre, University of British Columbia, Vancouver, Canada; 3 University of Wisconsin Carbone Comprehensive Cancer Center, Highland Ave, Madison, Wisconsin, United States of America; 4 Department of Pathology University of Wisconsin School of Medicine and Public Health, Highland Ave, Madison, Wisconsin, United States of America; 5 Environmental and Molecular Toxicology, University of Wisconsin, University Ave Madison, Wisconsin, United States of America; University of Kentucky College of Medicine, UNITED STATES

## Abstract

**Purpose:**

Androgen deprivation therapy (ADT) commonly leads to incomplete cell death and the fate of persistent cells involves, in part, a senescent phenotype. Senescence is terminal growth arrest in response to cell stress that is characterized by increased lysosomal-β-galactosidase (GLB1) the origin of senescence associated-β-gal activity (SA-β-gal). In the current study senescence is examined *in vivo* after ADT use in a neoadjuvant trial.

**Methods and materials:**

Tissue microarrays were generated from prostate cancer specimens (n = 126) from a multicenter neoadjuvant ADT trial. Arrays were subjected to multiplexed immunofluorescent staining for GLB1, Ki67, cleaved caspase 3 (CC3) and E-cadherin. Automated quantitative imaging was performed using Vectra™ and expression correlated with clinicopathologic features.

**Results:**

Tissue was analyzed from 59 patients treated with neoadjuvant ADT and 67 receiving no therapy preoperatively. Median follow-up was 85.3 mo and median ADT treatment was 5 mo. In PC treated with neoadjuvant ADT, GLB1 expression increased in intermediate Gleason score (GS 6–7; p = 0.001), but not high grade (GS 8–10) cancer. Significantly higher levels of GLB1 were seen in tissues undergoing neoadjuvant ADT longer than 5 months compared to untreated tissues (p = 0.002). In contrast, apoptosis significantly increased earlier (1–4 mo) after ADT treatment (p<0.5).

**Conclusions:**

Increased GLB1 after neoadjuvant ADT occurs primarily among more clinically favorable intermediate grade cancers and enrichment of the phenotype occurs in a temporally prolonged fashion. Senescence may explain the persistence of PCa cells after ADT. Given concerns for the detrimental longer term presence of senescent cells, targeting these cells for removal may improve outcomes.

## Introduction

Prostate cancer (PCa) thrives on circulating androgens and the initial step in managing advanced PCa is androgen deprivation therapy (ADT). While ADT remains an effective early treatment with 90% of patients demonstrating a response, the rate at which the majority of men will progress over several years to castrate-resistant prostate cancer (CRPC) varies. In advanced disease, survival after ADT is 60 months in modern populations.[1, 2] The persistence of dormant cancer cells after ADT remains an incompletely understood phenomenon that may lend important insight into failure after treatment.

Clinicopathologic factors available at the time of diagnosis incompletely inform the physician regarding progression rates after ADT. Prostate specific antigen (PSA) nadir, stage and Gleason grade are important determinates of treatment response. In patients with locally advanced and metastatic PCa treated with ADT the risk of androgen insensitivity within 24 months of treatment was 15-times higher if a PSA nadir was not achieved.[3] Rising PSA is one of the earliest signs of progression. [4–6] The risk of progressing to CRPC increases 5-fold with every point increase in Gleason score.[3, 7, 8] In SWOG 9994, factors that predicted longer survival at the time of diagnosis included minimal disease, better performance status, lack of bone pain, lower Gleason score, and lower PSA level.[8] However, substantial interpatient variation in responses were noted in this trial, and only 13% of patients with longer-term survival were accurately predicted emphasizing limitations to using these clinicopathologic factors.[9]

The molecular mechanisms and cellular changes that underlie the response of PCa to ADT are incompletely defined. A subset of androgen responsive PCa cells undergoes apoptosis in response to ADT.[10, 11] In CWR22 xenograft tumors apoptosis peaks 48hr after castration then rapidly decreases.[11] However, patient tumors after ADT show regression and decreased proliferation, but demonstrate infrequent apoptosis.[10, 12] Our laboratory was among the first to demonstrate that androgen withdrawal invokes a cellular senescence in prostate tumors.[13, 14] Cellular senescence is a terminal phenotype whereby cancer cells exposed to sub-toxic chemotherapeutic doses or other cellular stress undergo proliferative arrest and exit the cell cycle directly.[15] Senescence is distinct from autophagy a catabolic process involving the degradation of a cell's own components through the lysosomal machinery with a unique biomarker profile that also permits cell survival under stress.[16] Increased cyclin-dependent kinase inhibitors p27 and p16 are expressed in senescence, proteins elaborated at increased levels after ADT.[17] The most characteristic biomarker for determining cellular senescence are amplified levels of senescent-associated β-galactosidase (SA β-gal) activity detectable in frozen or fresh tissues.[18] This requirement for fresh tissues was recently circumvented by the development of a GLB1 antibody against SA β-gal that acts as a surrogate for identifying senescence in formalin-fixed paraffin-embedded tissues, thus permitting assessment of senescence biology in fixed tissues.[19]

Given its role as a tumor suppressor in aging and interest as a response to chemotherapy, it is hypothesized that senescence may be a prognostic marker of treatment responses.[13] Using a database of patients treated with neoadjuvant ADT followed by radical prostatectomy (RP), markers for senescence, apoptosis, and cell proliferation were analyzed on tissue microarrays to determine response to ADT. Senescence appears to explain the persistence of some PCa cells after ADT in tumors and may be a component in predicting response to therapy.

## Materials and methods

This study was performed under separate University of Wisconsin, Madison WI and University of British Columbia, Vancouver BC Institutional Review Board approvals. Tissue microarrays were utilized from patients undergoing ADT prior to radical prostatectomy that have been previously described.[20, 21] Data consisted of 2 separate tissue microarrays from PCa patients collected between 2000 and 2013 of which 94 were control and 59 received ADT. Length of neoadjuvant ADT treatment was 0-3mo (13pt), 4–5 (1pt), 6-9mo (36pt), 10-12mo (3pt) with a mean of 6.1mo (±2.9). Patients were divided into short (0-5mo) and extended (6-12mo) ADT treatment based on this mean. Clinicopathologic data was provided at diagnosis and at treatment for 59 ADT patients and 67 untreated patients.

Using automated Vectra™ system (Perkin Elmer,Waltham, MA), TMA slides were scanned and the levels of target biomarkers were measured as previously described in both cytoplasmic and nuclear compartments of the prostate epithelium and stroma.[19] Briefly, TMA sections were taken through routine deparaffinization and rehydration, HIER treatment with citrate for 20 min, and staining as previously described.[22] For quadruple multiplexed immunostaining, Ventana Discovery XT Staining Module was used. The TMA slides were stained with 4 primary antibodies including GLB1 (#ab55176; AbCam, Cambridge, MA, at 1:100); Ki67 (Abnova, Taipei, Taiwan, at 1:100), Caspase-3 (Asp175; Cell Signaling Technology, Danver, MA at 1:400) and E-cadherin antibodies,(Cell Signaling Technology, Beverly, MA at 1:100). Each primary antibody was detected with specific secondary antibodies and visualized with a unique BioCare Medical chromgen (Vulcan Fast Red, Bajoran Purple, DAB and Deep Space Black, respectively). E-cadherin antibodies were used to define the epithelial compartment for tissue segmentation.

For automated image acquisition and analysis, the stained slides were loaded onto the Vectra™ slide scanner and per-cell GLB1 target signals were quantitated for individual cores using the Vectra™ imaging system according to manufacturer’s protocol. InForm1.2 Software was used to segment tissue compartments (epithelium vs. stroma) and subcellular compartments (nucleus vs. cytoplasm). Both per cell and per core analyses were performed.

Cellular data was averaged to provide an expression level for each core. Individual cores were averaged for various groups and compared using Wilcoxon Mann-Whitney rank sum and Kruskall-Wallis H tests. Multiple cores from the same patient at identical time points were averaged to obtain an estimate of protein expression. GLB1 protein expression was compared in TMA specimens from patients who received neoadjuvant ADT against specimens from radical prostatectomy without prior therapy. The association of GLB1 levels with Gleason score, neoadjuvant treatment duration, margin status, and other pathological features were assessed. A p-value of <0.05 was considered statistically significant. Statistical analysis was performed using SPSS Version 21.0 (IBM).

## Results

Tissue microarrays constructed from RP tissues were obtained from a series of previously reported neoadjuvant ADT studies.[20, 21] Collectively, tissue was able to be analyzed from 59 patients treated with neoadjuvant ADT and 67 receiving no therapy preoperatively. Median follow-up for the entire study population was 85.3 mo. Clinicopathologic data available at diagnosis is provided Table 1. Patients treated with neoadjuvant ADT versus controls had higher initial PSAs (p<0.001), Gleason grade (p = 0.005), number of positive cores (p = 0.01) and clinical stage (p<0.001). All patients underwent local treatment with radical prostatectomy. The median treatment duration for ADT in the neoadjuvant group was 7 mo (IQR 6–8). After RP, final pathologic stage was more advanced in the neoadjuvant ADT versus control group (p = 0.01) Table 2.

**Table 1:** Clinicopathologic Data at Diagnosis.

	No ADT (n = 67)	ADT (n = 59)	p-value
**PSA**	6.5 (IQR 5.3–8.5)*	9.6 (IQR 6.7–15.6)	**0.0003**
**Positive Cores**	3 (IQR 2–4)*	4 (IQR 3–6)	**0.01**
**Gleason Score (all)**	7 (IQR 6–7)*	7 (IQR 7–8)	**0.005**
**5**	0	1 (1.7%)	**0.003 **
**6**	18 (28)	10 (17)	** **
**7**	40 (63)	28 (48)	** **
**8**	4 (6.3)	6 (10)	** **
**9**	2 (3.1)	14 (24)	** **
**Clinical Stage**			**< .0001**
**T1b**	1(1.6)	0	** **
**T1c**	23(36.5)	11(18.6)	** **
**T2a**	30(47.6)	18(30.5)	** **
**T2b**	5(7.9)	7(11.9)	** **
**T2c**	4(6.4)	8(13.6)	** **
**T3**	0	15(25.4)	** **
**Length of ADT (mo)**	-	6.1 (2.9)	

* Median PSA, positive cores, and Gleason Score.

**Table 2:** Pathologic Data after Radical Prostatectomy.

	No ADT (n = 67)	ADT (n = 59)	p-value
**Gleason Score (all)**	7 (IQR 7–7)	7 (IQR 7–8)**	0.41
**6**	15 (22%)	7 (22%)	0.44
**7**	42 (63)	17 (53)	
**8**	5 (7.5)	2 (6.3)	
**9**	5 (7.5)	6 (18.8)	
**Positive Margin**	17 (25)	12 (20)	0.5
**Positive Lymph Node**	0	3 (5.7)	0.25
**Pathologic Stage**			**0.01**
**T2**	47 (70)	28 (47)	** **
**T3**	20 (30)	30 (51)	** **
**T4**	0	1 (1.6)	** **

* Median Gleason score.

** Gleason scoring is inaccurate post ADT and only 32 samples were assigned a post-radical prostatetomy value.

**Fig 1:**
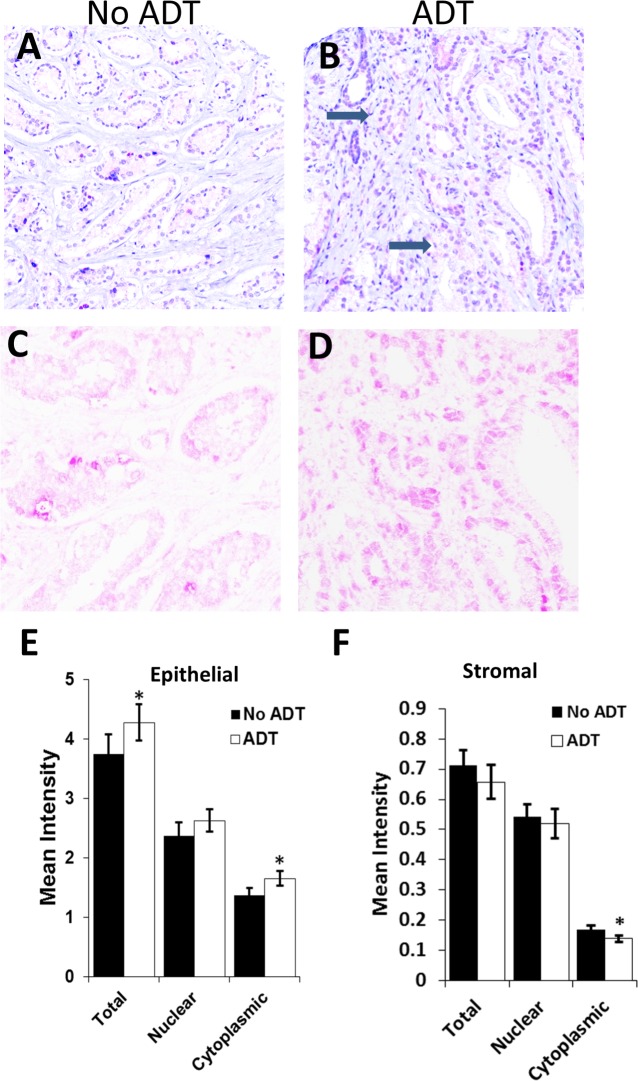
Higher GLB1 levels after neoadjuvant ADT in prostate cancer tumor tissues. Immunohistochemistry for GLB1 was performed on tissue arrays as described in the methods and staining quantitated automatically using VECTRA. Lower power H&E staining (20X) of control (A) and neoadjuvant (B) ADT treated intermediate grade prostate cancer tissues. Morphologic changes associated with ADT exposure include shrinkage/loss of acini (→) making Gleason scoring inapplicable. Increased levels of GLB1 (purple) are shown in nuclear and cytoplasmic compartments of ADT treated (D) and untreated (C) epithelium under higher power. (E) Epithelial (tumor) levels of GLB1 were significantly increased in ADT treated specimens overall and in the cytoplasm. (F) No significant increases in GLB1 expression in stromal tissue treated with ADT compared to untreated tissues. *p<0.05; mean±SE.

Immunohistochemistry for GLB1 was performed, quantitated, and analyzed utilizing Vectra™, an automated imaging system, within the epithelial and the stromal compartments of the ADT-treated (54) and control final surgical specimens (94).[19] Histologically PCa after ADT displayed compressed, fused glands, and cells contain abundant clear vacuolated cytoplasm and shrunken nucleoli in some areas when compared to control tissues (Fig 1A, 1B, 1C and 1D). In the epithelial (tumor) compartment (Fig 1E), GLB1 staining was expressed at higher levels, mean intensity 3.6 vs 4.3 (p = 0.02) in ADT-exposed prostate samples compared to controls. Stromal compartment staining revealed GLB1 expression was roughly 18% of epithelial levels and showed no significant change with ADT (Fig 1F). The majority of senescent-associated β-galactosidase (SA β-gal) activity originated from the endoplasmic reticulum present in the cytoplasm and this component was subsequently utilized for further analyses of the tumor epithelium.[18]

**Fig 2:**
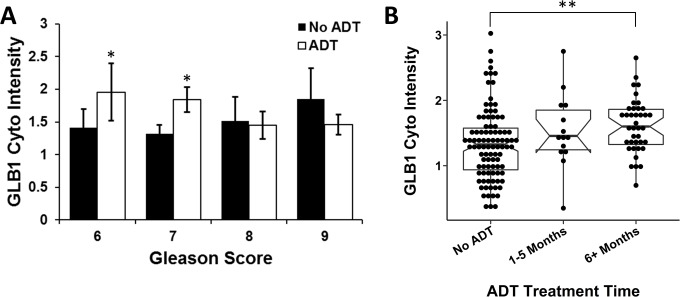
GLB1 levels increase after ADT in intermediate grade PCa and over time. (A) GLB1 expression was measured in radical prostatectomy specimens categorized by ADT status and pretreatment Gleason grade. Higher levels of GLB1 were expressed in intermediate PCa tissues treated with ADT. GLB1 after ADT was not increased in high grade cancers (Gleason 8 + 9). (B) GLB1 expression was evaluated over neoadjuvant ADT treatment duration. PCa tissues exposed to ADT longer than 5 months expressed significantly higher levels of GLB1 compared to no ADT, but shorter durations of ADT exposure did not reach significance. (*p<0.05;**p<0.01; mean±SE).

GLB1 levels on the final RP specimens, both control and ADT treated, were initially analyzed for an association with preoperative biopsy Gleason score. GLB1 levels were significantly induced in Gleason score 6 (p = 0.02) and 7 (p = 0.01) tumors undergoing ADT therapy compared to no ADT (Fig 2A). No induction of GLB1 was observed in biopsy Gleason score 8 and 9 specimens comparing treated or untreated specimens. Grouping Gleason scores into intermediate (Gleason 6–7) and high grade (Gleason 8–10) PCa confirmed this induction of GLB1 after ADT occurred primarily in the intermediate 6 and 7 group (p = 0.001). Length of ADT exposure between the 2 groups did not significantly differ (intermediate 5.8 ±2.9 vs high 6.7 ± 2.5 mo; p = 0.3). A wide range of GLB1 expression was noted in intermediate grade tumors suggesting variable responses.

We examined whether there was any association between GLB1 expression under ADT treatment and adverse pathologic features. In ADT treated specimens, decreased GLB1 showed a nonsignificant trend towards an association with increased risk of positive margins (OR 0.24 95% 0.039 CI 1.5; p = 0.1). No association with PSA recurrence was noted (p = 0.75). For the intermediate grade ADT subgroup alone, decreased GLB1 was associated with a trend towards increased positive margins (OR 0.15 95%CI 0.015–1.485 p = 0.1).

The induction of the senescence phenotype and accumulation of these cells increases with time *in vitro*, but this interval is not known in many tissues including the prostate.[15] GLB1 levels were analyzed by duration of ADT treatment prior to prostate removal and compared to RP specimens not treated with ADT (Fig 2B). Higher levels of cytoplasmic GLB1 (p = 0.002) were found in neoadjuvant tissue treated ≥ 6 mo compared to untreated tissues. The average treatment time of ADT for the population overall was 6.1 ±2.9 mo with the majority of patients being treated for shorter than 3mo. The induction of senescence at ≥6 mo of ADT occurred primarily in intermediate Gleason score cancers compared to higher grade (p = 0.004). A wide range of GLB1 expression occurred in months 1–5 suggesting a range of tumor responses to ADT (Fig 2B).

**Fig 3:**
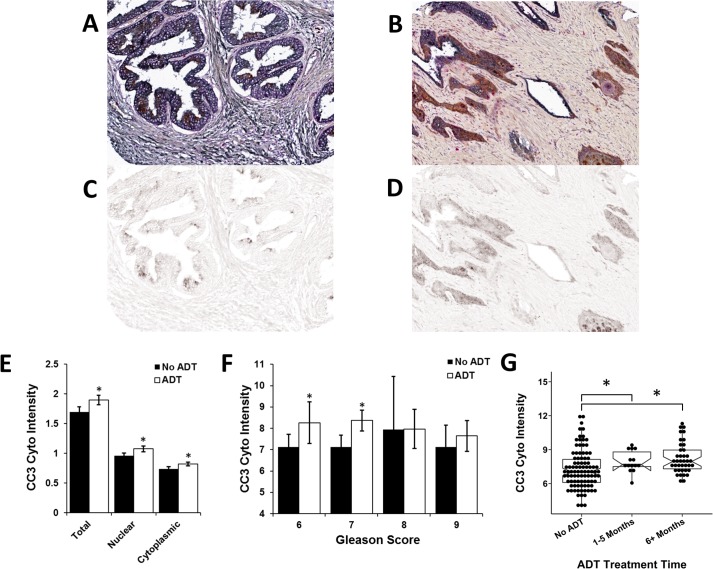
Cleaved caspase 3 (CC3) expression increases in intermediate grade cancers and occurs early after ADT. CC3 immunohistochemistry was performed on neoadjuvant ADT and control prostate tissues and then quantitated using the automated imaging analyzer VECTRA. Gleason scoring was performed on pretreatment biopsies. (A) Cell nuclear and cytoplasmic levels of CC3 demonstrate increased expression in ADT treated PCa tissues and increased levels in Gleason score 6 and 7 (B) compared to untreated controls. (C) CC3 expression is shown according to duration of neoadjuvant ADT treatment and compared to untreated controls. Significant increases in CC3 levels were seen within 1-5mo after ADT initiation. *p<0.05; mean±SE.

Apoptosis also results from ADT in prostate cells and a common pathway marker for this phenotype is Cleaved Caspase 3 (CC3). In concurrent staining, ADT increased expression of CC3 (Fig 3A) and this effect was primarily found in intermediate grade PCa (Gleason scores 6–7) (Fig 3B). CC3 staining significantly increased early (1–4 mo) after ADT treatment (Fig 3C). No correlations between GLB1 and CC3 or the Ki67 index was noted within individual tumors (data not shown).

## Discussion

Improving the ability to identify and treat patients that are unlikely to achieve a long-term response to ADT is an important goal. This is especially notable given recent results from the Phase III CHAARTED trial (ChemoHormonal Therapy Versus Androgen Ablation Randomized Trial for Extensive Disease in Prostate Cancer) through the Eastern Cooperative Oncology Group that demonstrated upfront chemotherapy with docetaxel and ADT improves survival by 10.5 mo versus ADT alone (HR = 0.63; *P* = 0.0006) in hormone naïve patients.[23] GLB1 has previously been demonstrated as a marker for PCa cell senescence and is induced in androgen-sensitive PCa cells after ADT *in vitro* and in animal models.[13, 19] In the current study, we demonstrate for the first time in human PCa that senescence occurs after ADT, associates with more favorable intermediate grade cancers, and accumulates in PCa tissues over months. Senescence appears to explain the persistence of some PCa cells after ADT in tumors and may be a component in predicting the response to therapy in some tumors.

Prior reports have indicated senescence develops after androgen deprivation *in vitro*. [13, 14] In the current study, the first to examine senescence in FFPE tissues after neoadjuvant ADT, GLB1 a protein product of the B-galactosidase gene[24] was quantitated in RP specimens and compared to preoperative variables and outcomes. GLB1 expression was dependent on duration of ADT and Gleason grade (Fig 1). Senescence *in vitro* takes >7 days after exposure to a senescence-inducing agent such as doxorubicin[15], but appears to develop only after longer exposure to ADT[13]. GLB1 levels increase in samples ≥6 months after ADT initiation. No samples were exposed to ADT for longer than 12 months in this dataset. Archival studies indicate maximal prostate volume regression after ADT typically takes >4 months.[25] In a previous study examining the metabolic activity of the prostate using MRI imaging with spectroscopy and dynamic contrast enhancement after ADT, a decrease in activity over several months was noted and complete at 6-months.[26] Similarly, the current data suggests that senescent cells accumulate over a prolonged time in PCa after ADT.

Our data shows that GLB1 expression after ADT depends on the Gleason score of the cancer in that significant induction of GLB1 was only noted in intermediate PCa and not in high grade disease (Fig 2). It is known that grade dictates, in part, the duration of ADT responses with Gleason 8–10 tumors having shorter periods of regression after ADT. [3] Therefore, the demonstration of robust senescence induction in intermediate grade tumors is consistent with a role for senescence as a tumor suppressor and may be a putative marker of more favorable tumor outcomes.[27] We did note a wide range in GLB expression in intermediate grade samples after ADT (Fig 2B) suggesting variable tumor responses, and associations between lower GLB1 and increased risk of positive surgical margins were suggested (p = 0.1). A comparison of final Gleason score to GLB1 staining on the RP specimens was not performed due to the finding that Gleason score readings can be spuriously elevated in hormonally-treated tissues.[28, 29]

The presence of increased senescence has been associated with improved outcomes and chemotherapy responses in colon cancer.[27] An elevated senescence index, based on the expression of p-ERK, HP1*γ* and PAI-1, all markers linked to senescence, correlated with better treatment responses to chemotherapy for stage IV colorectal cancer and longer progression-free survival (median 12 months vs 6 months, p = 0.04). Senescent tumor marker expression has previously been associated with improved prognosis and lower rates of relapse following treatment in other tumors.[30] Other important senescent markers include the cyclin-dependent kinases inhibitors p16 and p27 that demonstrate increased expression when cells growth arrest, but are frequently inactivated in tumors and not specific to the senescent phenotype.[15, 31] Paraffin-embedded markers for senescence has made widespread evaluation difficult. We recently confirmed GLB1 as a marker of senescence in a well-described reference cell culture system and applied this to PCa tissue samples.[19] In that study of FFPE prostate tissues, GLB1 levels were markedly increased in HGPIN (p<0.0001), a pathologic entity known to contain senescent cells[32] and further found that primary PCa samples associated with metastatic disease had lower levels of expression compared to localized tumors (p = 0.0003). No significant correlation with Gleason score was noted in these untreated primary PCa samples. These previous data connect GLB1 as a marker for improved outcomes in PCa.

Our study has several limitations. First, our study relied on tissue microarrays from selected patients at academic centers that were treated with neoadjuvant ADT, a group potentially not reflecting larger population-based PCa outcomes. Secondly, due to limitations in the patient population, although one of the largest collections available, we were unable to definitively investigate ultimate outcome variables such as cancer death. Finally, tissue arrays may not reflect the biology of all tumors in the prostate specimen given the known heterogeneity of cancers seen in RP specimens.

These data also have implications in the management of progressive PCa. Persistent senescent tumor cells after ADT have potential tumor-promoting effects via a senescence-associated secretory phenotype that may play a role in the progression to CRPC.[14] Nevertheless, it is possible that persistent senescent cells seen after ADT may present an ‘Achilles Heel’ in the treatment of residual cancer. Following senescence induction, a recent study by Dorr and colleagues found that senescent cancer cells uniformly demonstrate increased metabolic activity and elevated levels of proteolytic stress that when combined with glycolysis blocking agents or proteolytic inhibitors resulted in cell lethality.[33] Identifying senescent cells, an objective of GLB1 staining, and targeting these cells for apoptosis in a synthetic lethal approach may be a valid approach in the management of PCa with further study.

## Conclusions

In this first assessment of senescence after ADT in prostate cancer, we find senescence to be induced preferentially in intermediate grade versus high grade cancer and that GLB1 accumulates over time consistent with a delayed entry of cells into senescence. Senescence appears to explain the persistence of some PCa cells after ADT in tumors. Given concerns over the detrimental longer-term presence of senescent cells, targeting senescent cells for removal may improve outcomes.

## Supporting information

S1 Dataset Individual data for figures. (XLSX)
